# Supplementary material for: Sex differences in heart mitochondria regulate diastolic dysfunction
Source: Nat Commun. 2022 Jul 4;13:3850. doi: 10.1038/s41467-022-31544-5 (PMC9253085; doi:10.1038/s41467-022-31544-5)
Supplement: Supplementary file 1 — Supplementary figures [file 41467_2022_31544_MOESM1_ESM.pdf]

Supplementary Figure 1

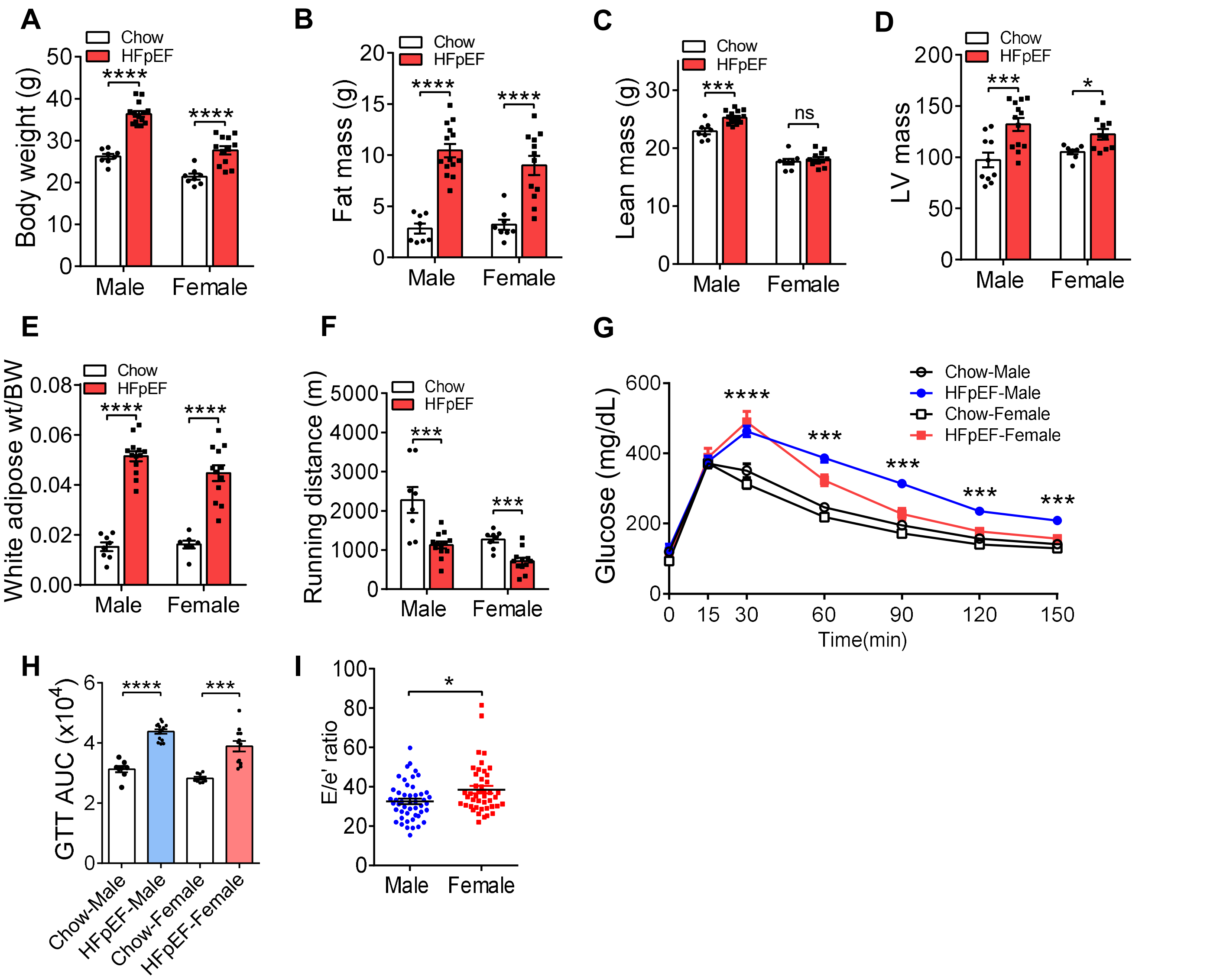

Supplementary Figure 1. Development of HFpEF in male and female mice.
A-F. Body weight (**A**), fat mass (**B**), lean mass (**C**), left ventricle mass (**D**), white adipose weight/body weight (**E**) and running distance (**F**) of C57BL/6J male and female mice after 7 weeks of chow diet and HFD + l-NAME diet. Male-chow, n = 8 (**A-C,E-F**) or 10 (**D**); male-HFpEF, n = 14 (**A-C**) or 13 (**D-F**); female-chow, n = 8; female-HFpEF, n = 12 (**A-C, E-F**) or 10 (**D**). **A**, p (male) < 0.0001; p (female) < 0.0001. **B**, p (male) < 0.0001; p (female) < 0.0001. **C**, p (male) = 0.0008. **D**, p (male) = 0.0006; p (female) = 0.011. **E**, p (male) < 0.0001; p (female) < 0.0001. **F**, p (male) = 0.0001; p (female) = 0.0003.
G-H. Glucose tolerance test (**G**) and area under curve (**H**) of C57BL/6J male and female mice after 7 weeks of chow diet and HFD + l-NAME diet. In **G**, asterisks indicate comparison between Male-HFpEF and Male-Chow. P < 0.0001. Chow-male, n = 8; HFpEF-male, n = 14; Chow-female, n = 8; HFpEF-female, n = 12. **H**, p (male) < 0.0001; p (female) = 0.0001.
I. 30 inbred strains of male and female mice were subjected to HFD + l-NAME diet for 7 weeks and E/e' ratio was measured after the feeding. N = 1-3 per strain. P = 0.011.

Each point represents a mouse. All data are presented as the mean ± SEM. *P < 0.05, **P < 0.01, ***P < 0.001, and ****p < 0.0001, by 2-way ANOVA (**A-H**), or Student's t test (**I**). Source data are provided as a Source Data file.

Supplementary Figure 2

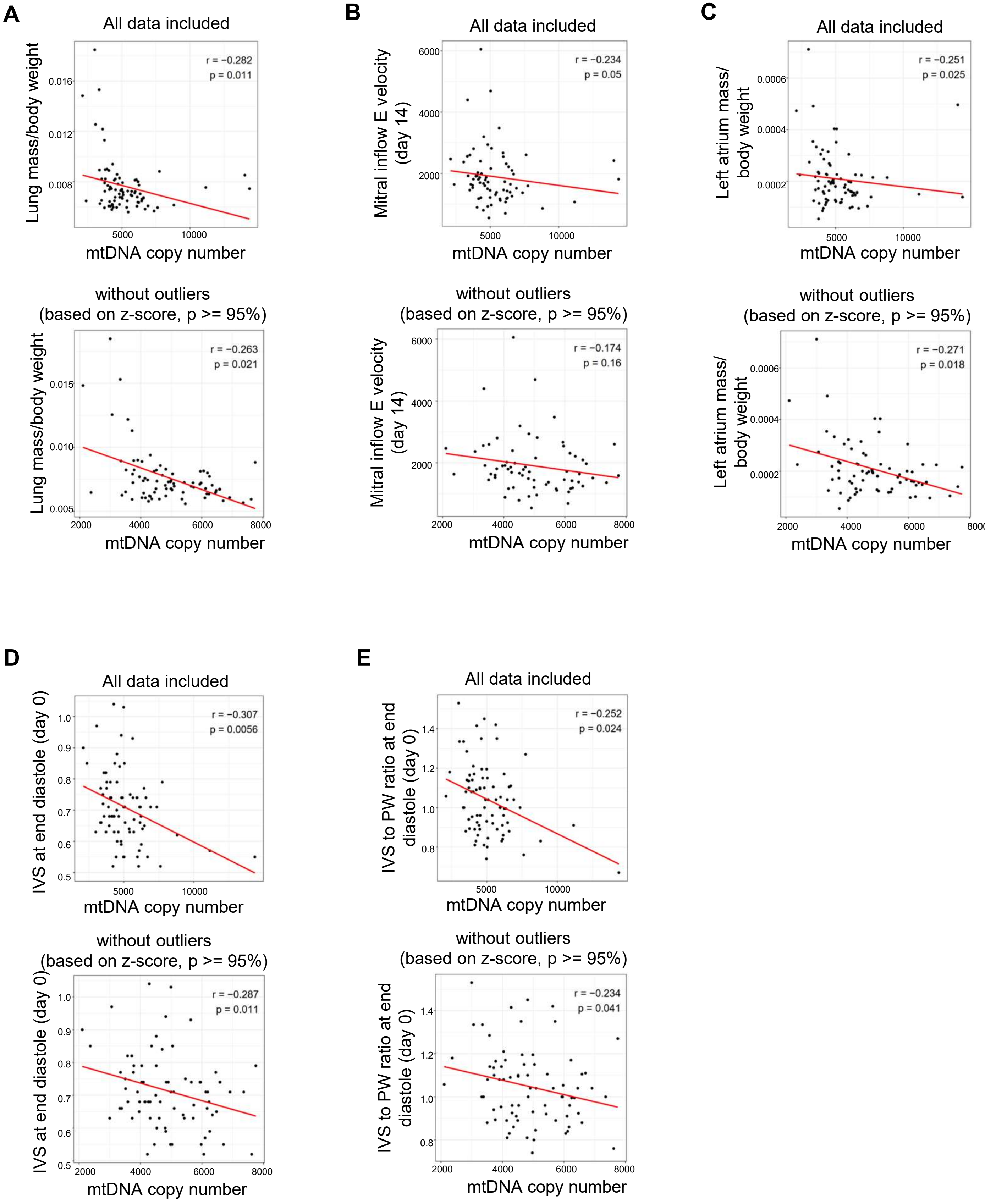

Supplementary Figure 2. mtDNA level was associated with traits of diastolic function.

A-E. mtDNA copy number correlated with lung mass/body weight (**A**), mitral inflow E velocity (day 14, **B**), left atrium mass/body weight (**C**), IVS at end diastole (day 0, **D**) and IVS to PW ratio at end diastole (day 0, **E**) in ISO-HMDP mice. Each point represents a mouse from an inbred strain. For each trait, all mtDNA content data included (top) and outliers excluded based on z-score (bottom) were shown. p-values are from biweight midcorrelation (bicor) tests.

Supplementary Figure 3

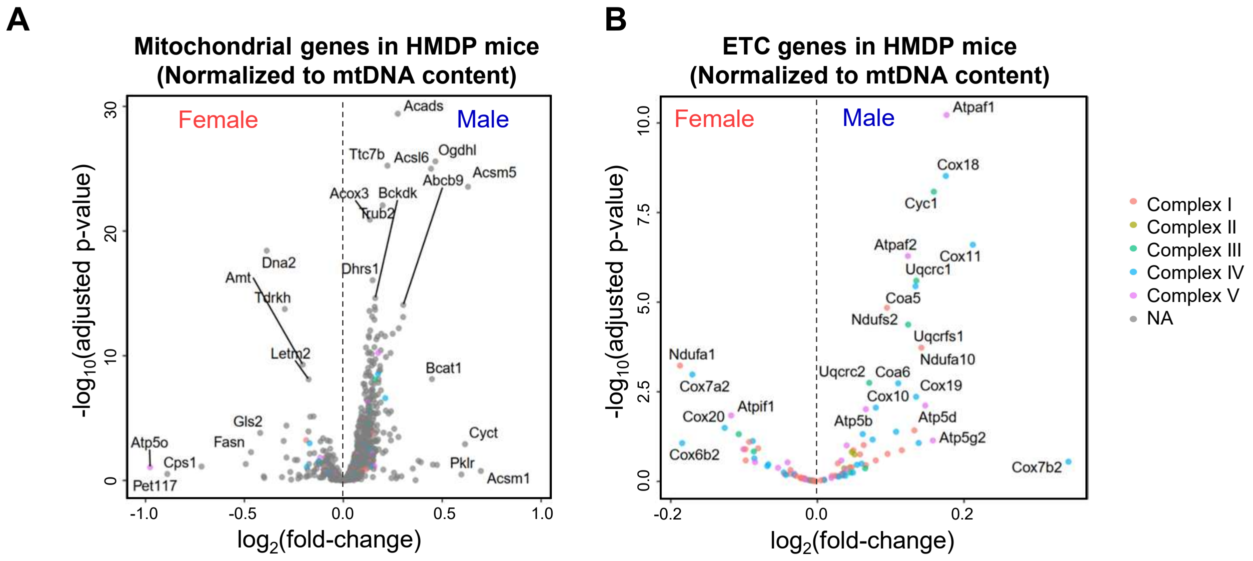

Supplementary Figure 3. Male mice exhibited higher mitochondrial gene expression compared to female mice.
A-B. Differential expressed mitochondrial genes (**A**) and electron transport chain (ETC) genes (**B**) in 100 strains of HMDP mice were normalized to mtDNA content in each strain. The gene expression values were strain-averaged. 77 strains for which male and female mice were sequenced were included. DESeq2 analysis was performed using mitochondrial DNA content as one of the terms in the experimental design. DESeq2 results use the Wald test, corrected for false discovery rate (FDR) using the Benjamini-Hochberg (BH) method.

Supplementary Figure 4

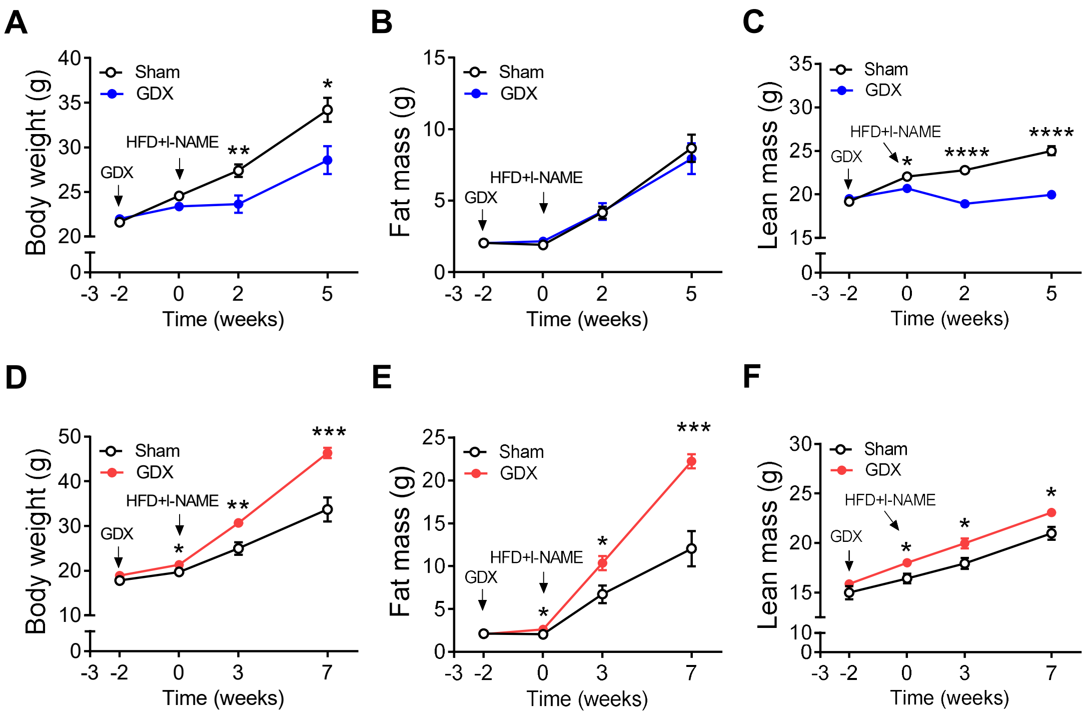

Supplementary Figure 4. Gonadectomy and HFpEF induction in C57BL/6J male and female mice.
A-C. Body weight (**A**), fat mass (**B**), and lean mass (**C**) of C57BL/6J male mice after gonadectomy and HFD + l-NAME diet. N = 13. **A**, p (week 2) = 0.004; p (week 5) = 0.011. **C**, p (week 0) = 0.014; p (week 2) < 0.0001; p (week 5) < 0.0001.
D-F. Body weight (**D**), fat mass (**E**), and lean mass (**F**) of C57BL/6J female mice after gonadectomy and HFD + l-NAME diet. Sham, n = 6; GDX, n = 7. **D**, p (week 0) = 0.034; p (week 2) = 0.002; p (week 5) = 0.0008. **E**, p (week 0) = 0.029; p (week 2) = 0.017; p (week 5) = 0.0004. **F**, p (week 0) = 0.011; p (week 2) = 0.019; p (week 5) = 0.014.

All data are presented as the mean ± SEM. *P < 0.05, **P < 0.01, ***P < 0.001, and ****p < 0.0001, by two-sided Student's t test. Source data are provided as a Source Data file.

Supplementary Figure 5

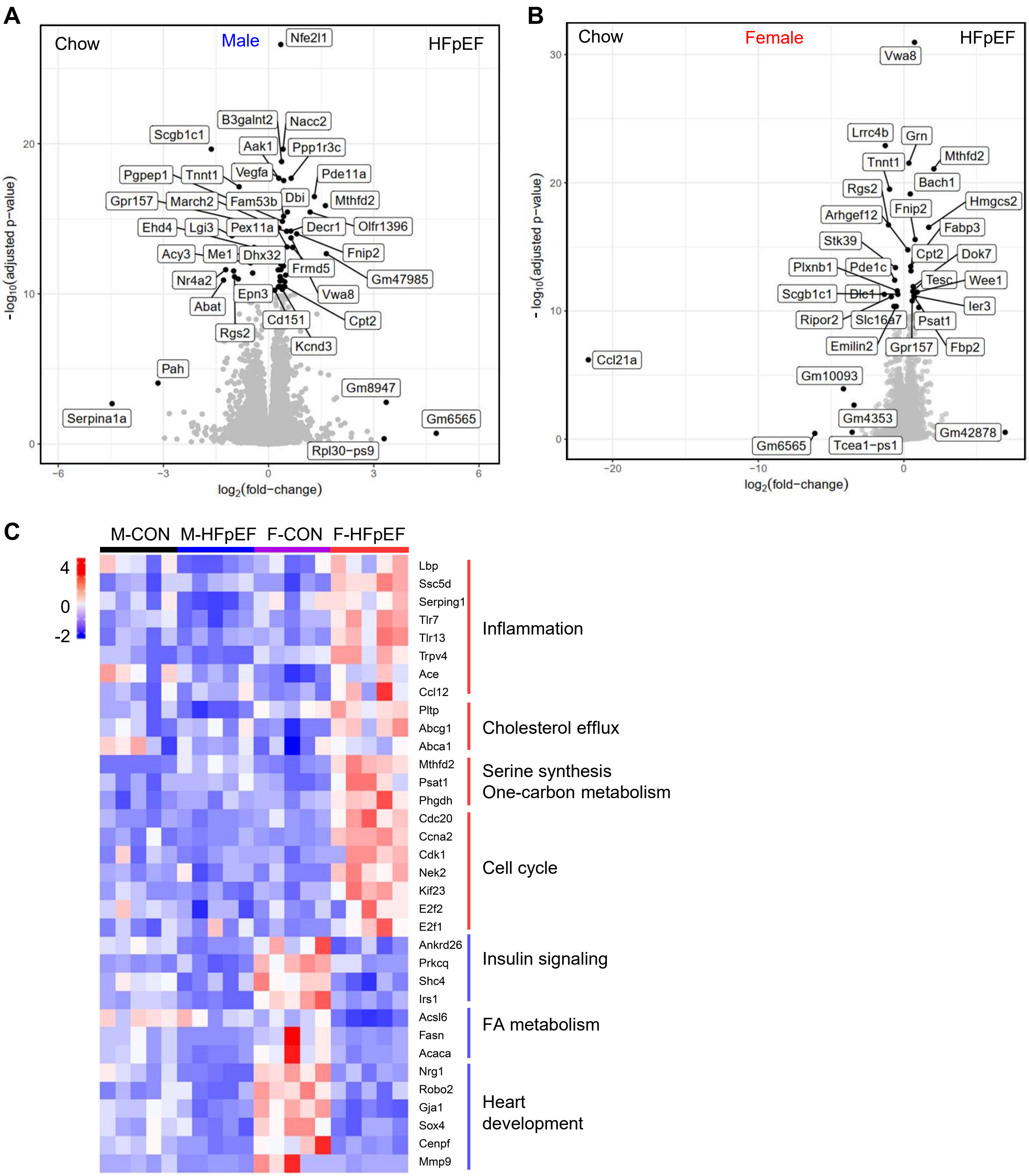

Supplementary Figure 5. Differential expressed genes in male and female hearts after HFpEF.
A-B. Volcano plot showing differential expression genes in male (**A**) and female (**B**) heart tissue after 7 weeks of HFD + l-NAME diet relative to chow diet. The x axis represents fold change in log2 scale of HFpEF versus chow while the y axis indicates -log10(adjusted p-value). Each point represents a gene. DESeq2 results use the Wald test, corrected for FDR using the BH method.
C. Differential expressed genes (DEGs) and related pathways in male and female hearts. M, male; F, female; CON, chow diet; HFpEF, HFD + l-NAME diet.

Supplementary Figure 6

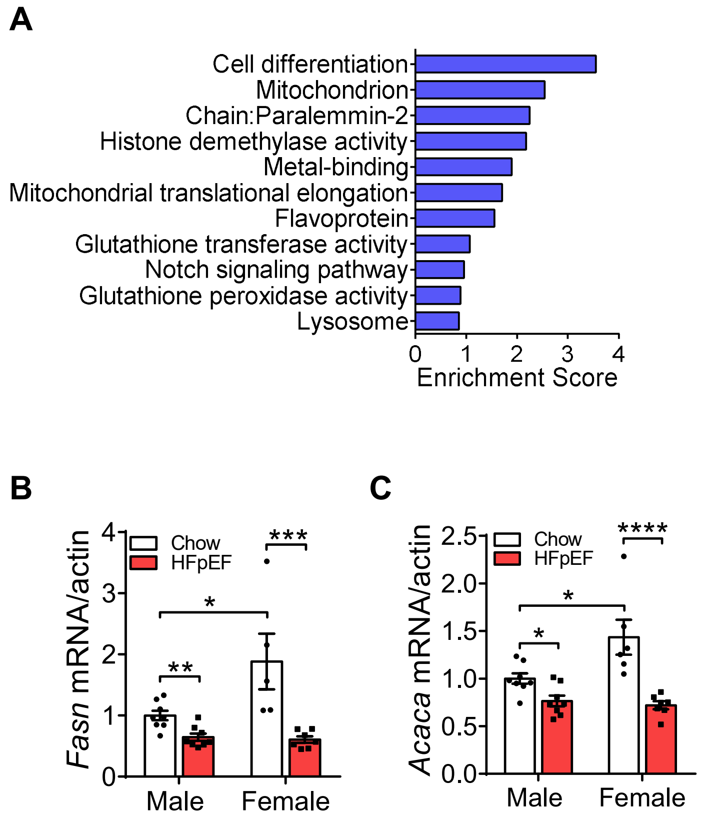

Supplementary Figure 6. Identification of *Acsl6* as a *cis*-regulator of diastolic function.
A. Enriched pathways showing genes that were differentially expressed in male and female patients with heart failure.
B-C. qRT-PCR showing mRNA levels of *Fasn* (**B**) and *Acaca* (**C**) in male and female hearts under Chow and HFpEF condition. Each point represents a mouse. All data are presented as the mean ± SEM. *P < 0.05, **P < 0.01 by 2-way ANOVA. Male, n = 8; female-chow, n= 5 (**B**) or 6 (**C**); female-HFpEF, n = 7. Source data are provided as a Source Data file. **B**, p (male) = 0.002; p (female) = 0.0003; p (Male-chow/Female-chow) = 0.01. **C**, p (male) = 0.01; p (female) < 0.0001; p (Male-chow/Female-chow) = 0.012.

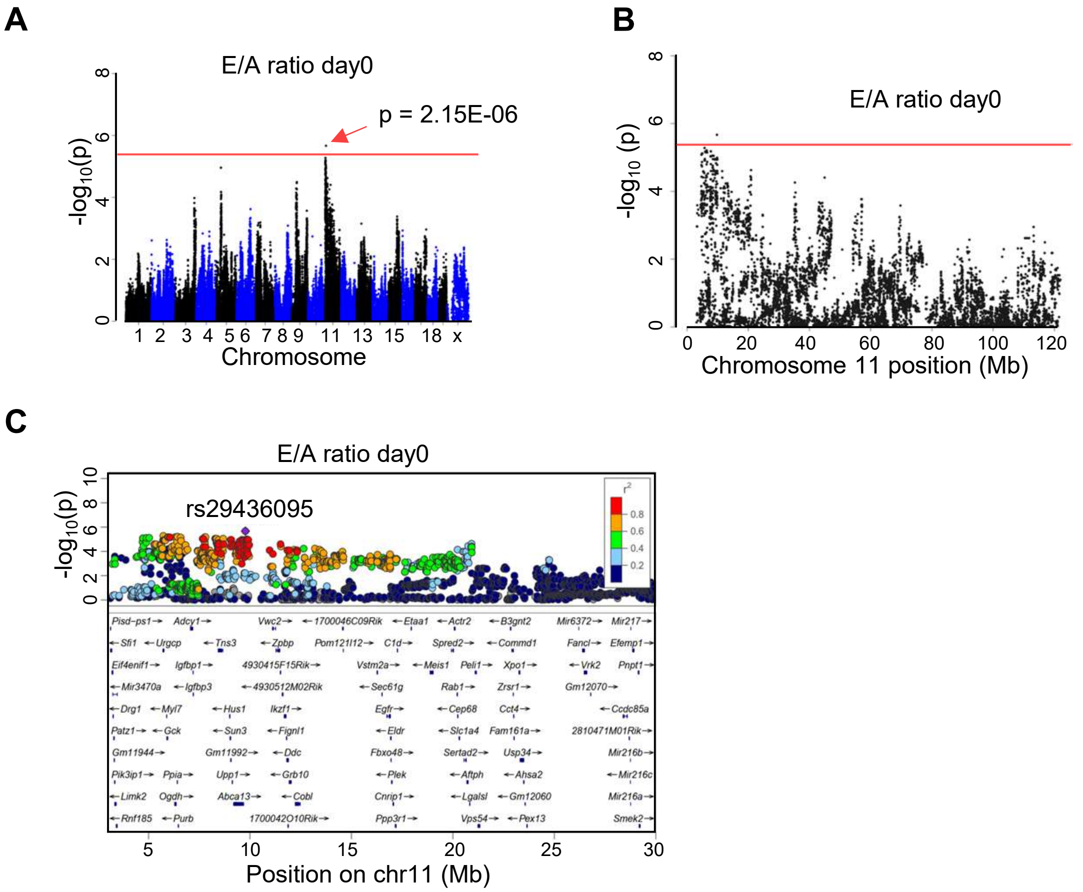

Supplementary Figure 7. GWAS of E/A ratio in female ISO-HMDP mice.

A. Manhattan plot showing the significance (-log10 of p) of all SNPs and GWAS of E/A ratio at baseline in female mice after 3 weeks of isoproterenol infusion (female ISO-HMDP).

B. Regional plots showing GWAS of E/A ratio at baseline and significance (-log10 of p) of all SNPs on chromosome 11. Association p-values from FaST-LMM are from Wald tests uncorrected for FDR (**A-B**).

C. LocusZoom plot for GWAS of E/A ratio at baseline and the nearby SNPs at chromosome 11.

Supplementary Figure 8

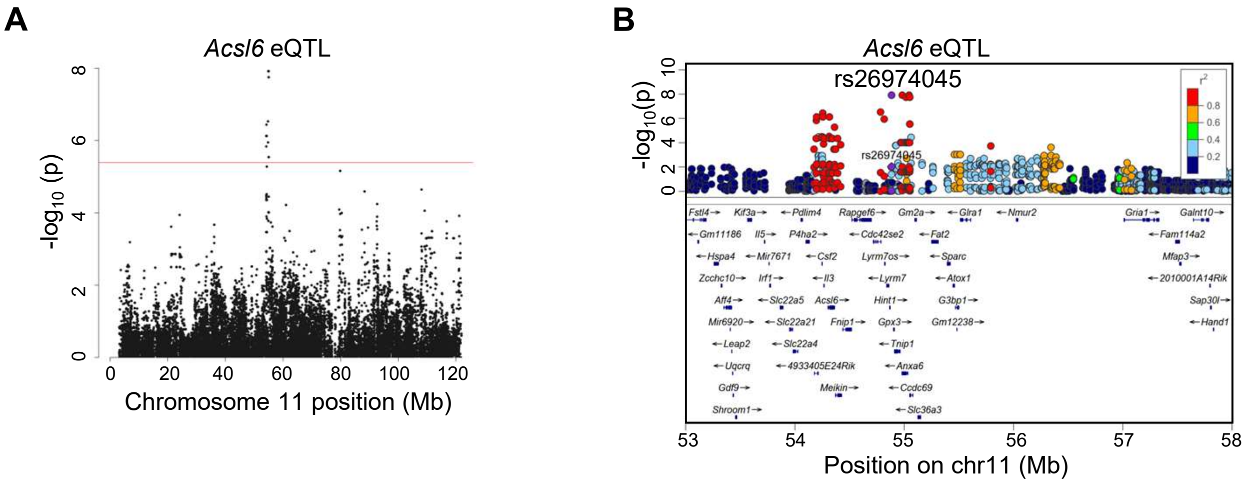

Supplementary Figure 8. Identification of *Acsl6* as a *cis*-regulator of diastolic function.

A. Regional plots showing heart expression QTL (eQTL) for *Acsl6* and significance (-log10 of p) of all SNPs on chromosome 11.

B. LocusZoom plot for *cis*-eQTL associations between *Acsl6* expression and the nearby SNPs at chromosome 11. Association p-values from FaST-LMM are from Wald tests uncorrected for FDR.

Supplementary Figure 9

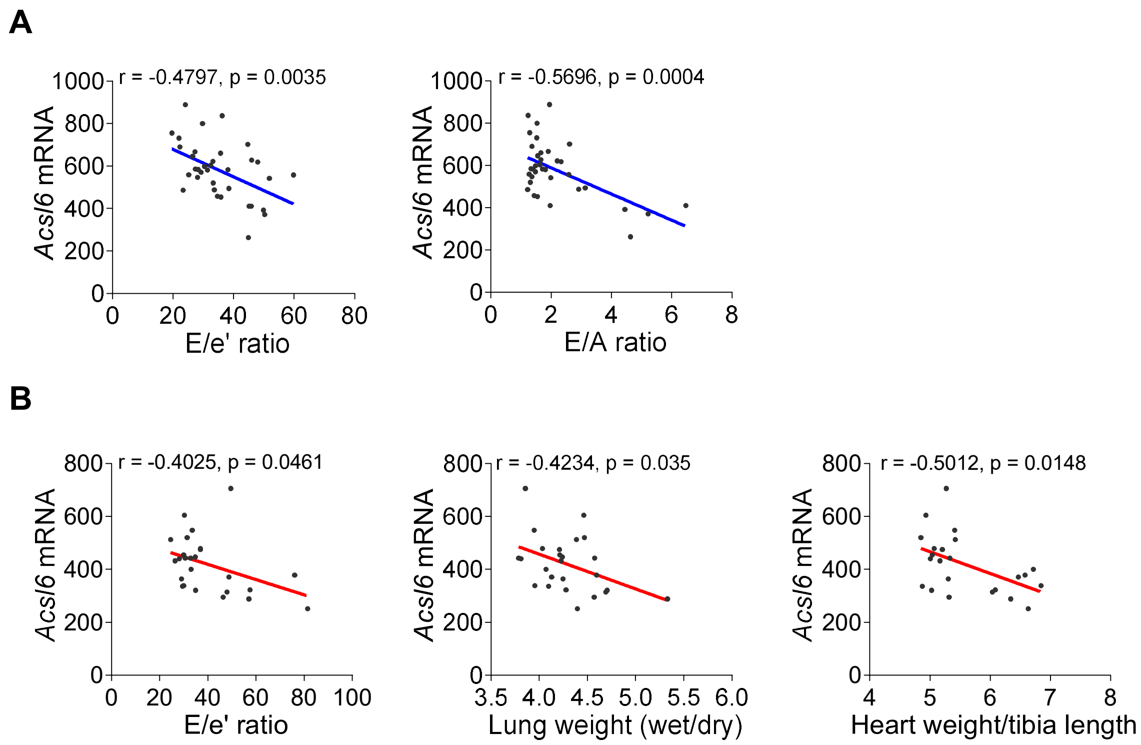

Supplementary Figure 9. *Acsl6* inversely correlated with diastolic dysfunction in mouse HFpEF model. **A-B.** *Acsl6* inversely correlated with parameters of diastolic function in C57BL/6J male (**A**) and female (**B**) mice fed with HFD + l-NAME. Each point represents a mouse. Source data are provided as a Source Data file. p-values are from biweight midcorrelation (bicor) tests.

Supplementary Figure 10

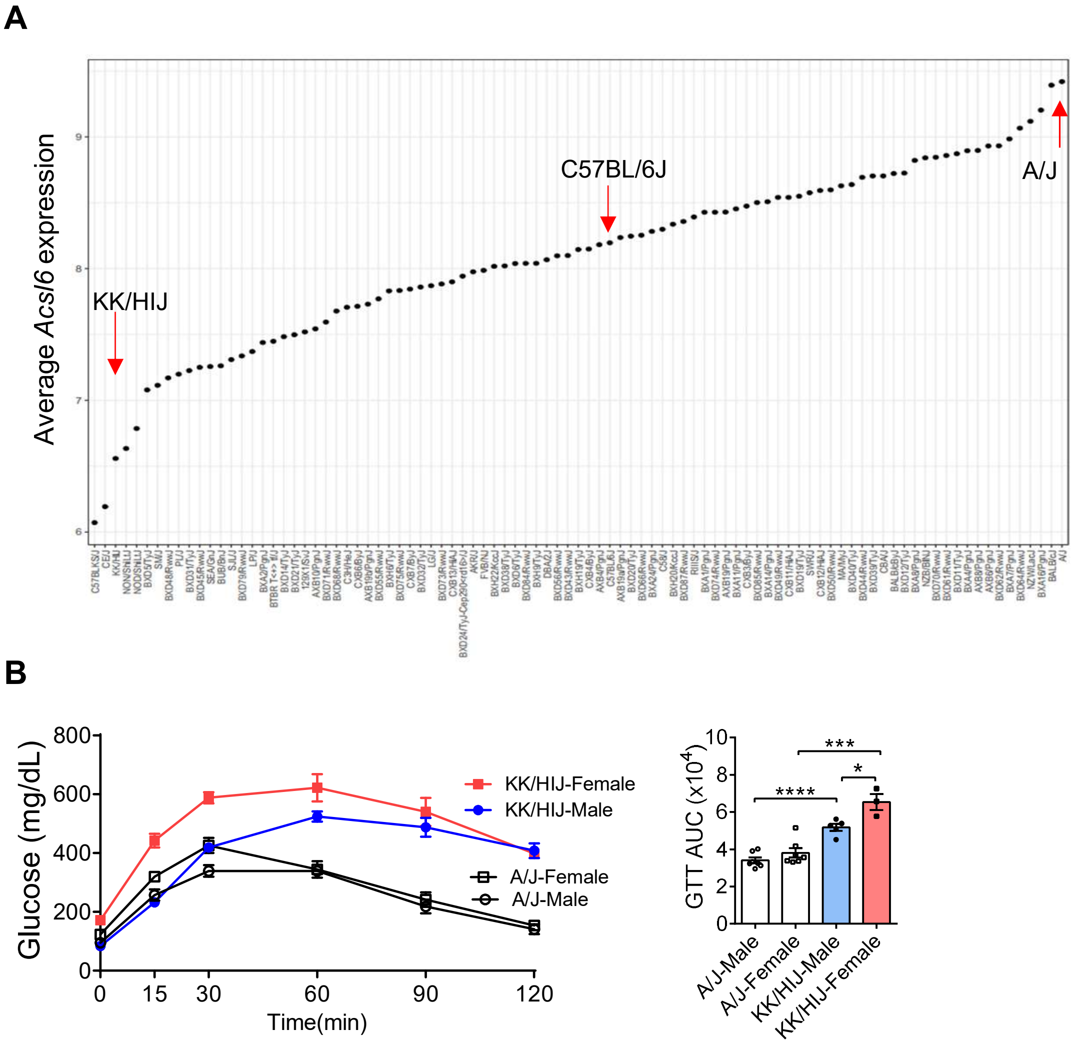

Supplementary Figure 10. *Acsl6* transcripts across female ISO-HMDP.
A. *Acsl6* expression was higher in strain A/J as compared with strain KK/HIJ, and strain C57BL/6J showed an intermediate *Acsl6* expression.
B. Glucose tolerance test and area under curve (AUC) in male and female A/J and KK/HIJ strains of mice after 7 weeks of HFD + l-NAME feeding. A/J, n = 7; KK/HIJ-male, n = 5; KK/HIJ-female, n = 3. p (A/J-Male/KK/HIJ-Male) < 0.0001; p (A/J-Female/KK/HIJ-Female) = 0.0004; p (KK/HIJ-Male/KK/HIJ-Female) = 0.014.

Each point represents a mouse. All data are presented as the mean ± SEM. *P < 0.05, ***P < 0.001, and ****P < 0.0001, by 2-way ANOVA (**B, right**). Source data are provided as a Source Data file.

Supplementary Figure 11

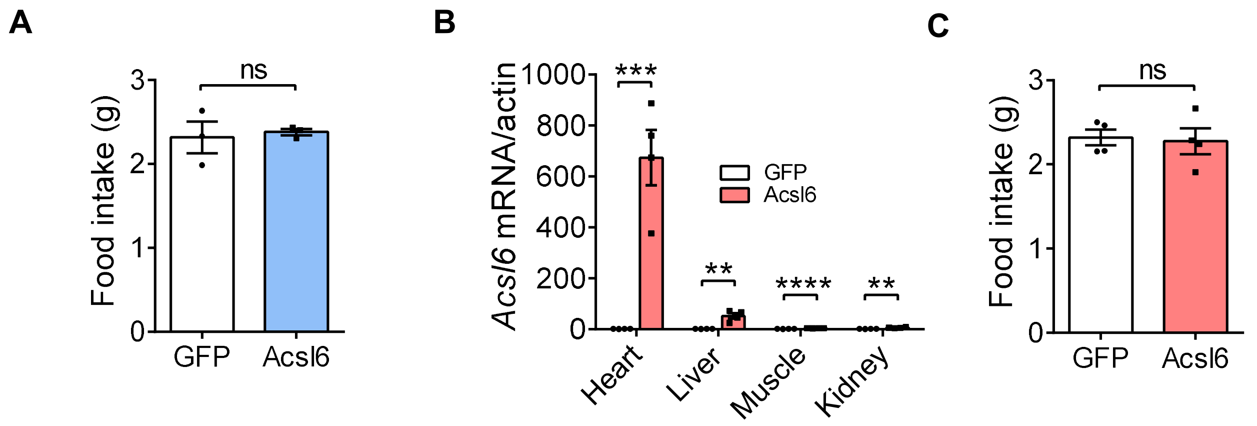

Supplementary Figure 11. Food intake and *Acsl6* expression in C57BL/6J male and female mice treated with AAV9 and 7 weeks of HFD + l-NAME feeding.

A. Food intake of C57BL/6J male mice treated with AAV9 and 7 weeks of HFD + l-NAME feeding. N = 3.

B. qRT-PCR showing relative levels of *Acsl6* mRNA in the heart, liver, skeletal muscle and kidney of C57BL/6J female mice treated with AAV9 and 7 weeks of HFD + l-NAME feeding. mRNA levels were normalized to actin. N = 4. p (heart) = 0.0008; p (liver) = 0.003; p (muscle) < 0.0001; p (kidney) = 0.005.

C. Food intake of C57BL/6J female mice treated with AAV9 and 7 weeks of HFD + l-NAME feeding. N = 4.

Each point represents a mouse. All data are presented as the mean ± SEM. ns, not significant. **P < 0.01, ***P < 0.001, and ****P < 0.0001, by two-sided Student’s t test. Source data are provided as a Source Data file.

Supplementary Table 1

Cardiac function of mice treated with AAV9-GFP or AAV9-Acsl6

Parameters	AAV9-GFP	AAV9-Acsl6	pvalue (Avsl6 vs. GFP)
Female-MV E/A baseline	1.479±0.075	1.384±0.115	0.214
Female-MV E/A 4 weeks	2.830±0.599	1.968±0.126	0.031
Female-MV E/A 7 weeks	3.172±0.921	2.123±0.400	0.082
Female-MV E/e' baseline	21.278±3.289	22.299±2.277	0.628
Female-MV E/e' 4 weeks	46.804±3.858	29.340±6.199	0.003
Female-MV E/e' 7 weeks	46.225±10.491	30.475±7.215	0.048
Female-EF baseline	60.179±1.958	56.663±2.860	0.089
Female-EF 4 weeks	55.728±3.442	60.846±6.769	0.226
Female-EF 7 weeks	54.010±2.49	57.381±3.371	0.159
Female-LV Mass baseline	93.071±6.981	87.767±7.702	0.347
Female-LV Mass 4 weeks	108.617±7.126	96.150±8.001	0.043
Female-LV Mass 7 weeks	111.719±12.640	97.737±2.333	0.068
Male-MV E/A 7 weeks	2.532±0.130	1.870±0.121	0.010
Male-MV E/e' 7 weeks	44.217±0.884	30.279±2.488	0.002
Male-EF 7 weeks	60.396±2.730	57.859±2.199	0.497
Male-LV Mass 7 weeks	129.672±5.453	111.602±2.793	0.026

p-values are from Student's t-tests uncorrected for multiple comparisons.
